# Monitoring for *Plasmodium falciparum *drug resistance to artemisinin and artesunate in Binh Phuoc Province, Vietnam: 1998-2009

**DOI:** 10.1186/1475-2875-9-181

**Published:** 2010-06-24

**Authors:** Ngo V Thanh, Tran Q Toan, Alan F Cowman, Gerard J Casey, Bui Q Phuc, Nong T Tien, Nguyen M Hung, Beverley-Ann Biggs

**Affiliations:** 1 National Institute of Malariology Parasitology and Entomology, Hanoi, Vietnam; 2 Walter and Eliza Hall Institute for Medical Research, Parkville, Victoria, 3050, Australia; 3 Department of Medicine (RMH/WH), The University of Melbourne, 4th Floor Clinical Sciences Building, The Royal Melbourne Hospital, Parkville, Victoria, 3050, Australia; 4 Victorian Infectious Diseases Service and Centre of Clinical Research Excellence in Infectious Diseases (CCREID), The Royal Melbourne Hospital, Parkville, Victoria, 3050, Australia

## Abstract

**Background:**

Artemisinin derivatives have been used for malaria treatment in Vietnam since 1989. Reported malaria cases have decreased from 1,672,000 with 4,650 deaths in 1991, to 91,635 with 43 deaths in 2006. Current national guidelines recommend artemisinin-based combination therapy (ACT), although artesunate is still available as monotherapy through the private sector. Recent reports suggest that effectiveness of ACT and artesunate monotherapy has declined in western Cambodia. This study examined *Plasmodium falciparum *resistance patterns over 10 years in southwest Vietnam in infected patients treated with artemisinin compounds.

**Methods:**

The study was conducted in two communes in Phuoc Long district, Binh Phuoc province, 100 km west of the Cambodian border. This was chosen as a likely site for emerging artemisinin resistance because of the high prevalence of *P. falciparum *malaria, and the length of time that artemisinin had been in use. In *vivo *and *in vitro *monitoring of *P. falciparum *susceptibility to anti-malarial drugs was conducted in 1998, 2001, 2004/5, and 2008/9. Patients with confirmed *P. falciparum *malaria received therapy with 5 or 7 days of artemisinin (1998 and 2001 respectively) or 7 days of artesunate

**Results:**

In the four surveys, 270 patients were recruited and treated. The mean parasite clearance times differed between 1998, 2001 and 2004/5 (1.8, 2.3 and 2.1 days, P < 0.01) but not between 1998 and 2008/2009. The mean parasite clearance times were correlated with parasite density at day 0 (r = 0.4; P < 0.001). Treatment failure rates after PCR adjustment were 13.8%, 2.9%, 1.2%, and 0% respectively. Susceptibility of *P. falciparum *to artemisinin in *in vitro *tests was stable during the period, except for a rise in EC90 and EC99 in 2001.

**Conclusions:**

This study showed stable levels of *P. falciparum *sensitivity to artemisinin compounds in the two sites over a ten-year period. The introduction of ACT in this area in 2003 may have protected against the development of artemisinin resistance. Adherence to the latest WHO and Vietnamese guidelines, which recommend ACT as first-line therapy in all malarious areas, and continued monitoring along the Vietnam-Cambodia border will be essential to prevent the spread of artemisinin resistance in Vietnam.

## Background

The global burden of malaria has increased during the last decade and in 2006 there were an estimated 247 million cases and 881,000 deaths worldwide [1]. Multi-drug resistance has increasingly become a major impediment to malaria control, although the discovery and evaluation of artemisinin in China in the l970's has strengthened global efforts to combat malaria. WHO now recommends that uncomplicated *P. falciparum *malaria be treated with artemisinin-based combination therapy (ACT) to prevent recrudescence and to delay the selection of resistant strains [2]. The emergence of artemisinin resistance would severely limit treatment options and recent reports from Cambodia confirm that this has already occurred in isolated locations [3-5].

In Vietnam, artemisinin derivatives have been used for malaria treatment since 1989. Reported malaria cases have diminished from 1,672,000 clinical cases with 4,650 deaths in 1991, to 91,635 with 43 deaths in 2006[6,7]. In spite of current national guidelines recommending ACT, artemisinin and artesunate are still available as monotherapy through the private sector (personal communication N. T. Tien). Self-treatment using anti-malarial drugs purchased from private pharmacies may result in inadequate treatment regimes and the use of poor quality medicines, practices that favour the emergence of drug resistance [8,9].

In 1998, the National Institute for Malariology, Parasitology and Entomology (NIMPE) and researchers at the University of Melbourne commenced monitoring in sentinel sites in Vietnam to examine drug resistance trends[10-12]. In Binh Phuoc province, the monitoring sites were located approximately 100 km from the Cambodian border. In this study, sensitivity to artemisinin and artesunate was investigated in two sites in Binh Phuoc province over 10 years. The *in vivo *and *in vitro *results are presented for drug resistance monitoring surveys in 1998, and 2001 in Phuoc Trung commune, and in 2004/5 and 2008/9 in Bu Gia Map commune.

## Methods

### Study sites

**Figure 1:**
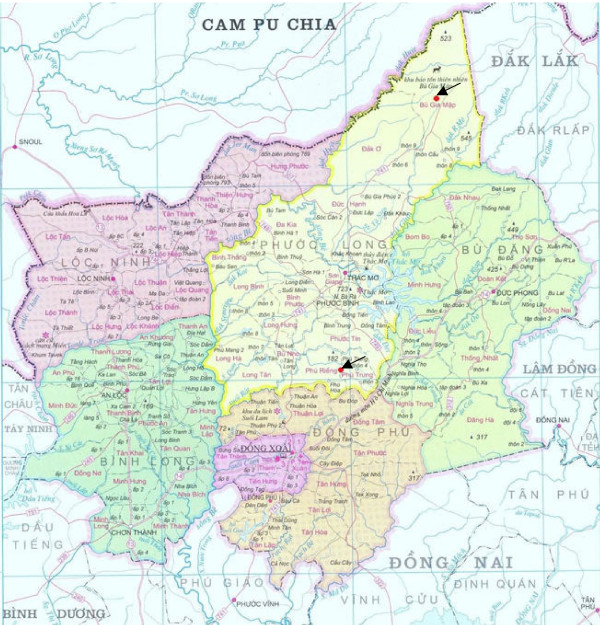
**Map of malaria drug resistance monitoring sites in Phuoc Long district, Binh Phuoc Province, Vietnam**. Arrows show sites in Phuoc Trung and Bu Gia Map communes. The border between Cambodia (Cam Pu Chia) and Vietnam is evident.

The study was conducted in Phuoc Long district, Binh Phuoc province, which is located 130 km north-east of Ho Chi Minh and 100 km west of the Cambodian border (Figure 1). This province has a high prevalence of malaria (> 10/1000 cases/year) with a peak season from September to December. It was the first province in Vietnam to use artemisinin in 1989. For the studies in 1998 and 2001 patients were recruited in Phuoc Trung commune and for the studies in 2004/5 and 2008/9, patients were recruited from Bu Gia Map commune. The distance between the two communes is about 80 kilometres, and both communes are located in Phuoc Long district. The National Malaria Control Programme first recommended the use of a locally produced dihydroartemisinin-based combination therapy, CV8 (CV8 contains 4 drugs: 32 mg dihydroartemisinin, 320 mg piperaquine phosphate, 5 mg primaquine phosphate and 90 mg trimethoprim) in these communes in 2003, and then changed the recommendation to dihydroartemisinin-piperaquine ('Artekin') in 2007. In 2008 and 2009, CV8 was phased out and Artekin became standard first-line treatment (N. T. Tien personal communication, National Malaria Treatment Guidelines, 2007, 2009).

### Recruitment of study participants

*In vivo *drug resistance monitoring surveys were performed in September to November 1998 and 2001, December 2004 to November 2005, and in September 2008 to March 2009, according to the WHO protocol current at the time [13,14]. It was expected that between 50 and 75 patients would be recruited in each survey.

Patients presenting to the commune health centre with uncomplicated *P. falciparum *malaria, who were aged three years and above and had asexual parasitaemia between 1,000 and 200,000 parasites per μl were invited to participate in the study. All participants 16 years and above, and carers of those under 16 years, provided written informed consent. Exclusion criteria included pregnancy, vomiting more than twice in the previous 24 hours, a convulsion in the previous 24 hours, or a history of allergy to artemisinin or derivatives. Patients who had received quinine, artemisinin or derivatives within the last seven days, 4-aminoquinolines within the last 14 days, pyrimethamine and/or sulphonamides within the last 28 days, or mefloquine within the last 56 days, were excluded from the study. The parasite clearance time was defined as the time from the initiation of therapy until the first negative blood film that remained negative for 48 hours.

Blood samples were collected from all *P. falciparum *positive cases before treatment, Blood smears were made each day until two consecutive slides were negative and on Day 7, 14, 21, 28, as well as from those who returned with malaria symptoms. Blood was drawn by venipuncture into a vacutainer tube containing EDTA. Thick and thin blood smears were examined after staining with 5% Giemsa. Parasitaemia was estimated by counting the number of asexual parasites per 1,000 leucocytes in the thick blood film, and estimates assumed a mean count of 8,000 leucocytes/μl.

### Treatment of malaria and follow-up

Participants were treated with artemisinin for 5 days in 1998 (20 mg/kg on Day 0, 10 mg/kg on Day 1-4) (Mediplantex, Hanoi, Vietnam), artemisinin for seven days in 2001 (20 mg/kg on Day 0, 10 mg/kg on Day 1-6) (Mediplantex, Hanoi, Vietnam), artesunate for seven days in 2004/5 (4 mg/kg on Day 0, 2 mg/kg on Day 1-6) (Guilin Pharma, Guilin, China; supplied by WHO), and artesunate for seven days in 2008/9 (4 mg/kg on Day 0, 2 mg/kg on Day 1-6, Naphaco, Nam Dinh province, Vietnam). Naphaco is a Vietnamese manufacturer who was GMP certified after inspection by WHO in 2008. Health clinic staff directly observed all treatment doses.

Patients were observed in the health clinic for the first seven days. If the patient vomited within 30 minutes after administration, the full dose was repeated; if vomiting occurred between 30 to 60 minutes after administration, half the dose was repeated. Follow-up appointments were scheduled for days 14, 21 and 28 or if they became unwell and consisted of a physical examination, a blood sample and completion of a standardised data collection form. If treatment failure occurred, patients were treated with CV8 after blood (50 μl × 2) was collected by finger prick and transferred onto 3 MM Whatman filter paper for DNA analysis.

Treatment failure was defined according to WHO guidelines [13].

### In vitro drug sensitivity assays

*In vitro *micro-test plates (WHO plates) containing chloroquine and mefloquine were prepared by Sain Penang University, Malaysia. Piperaquine, dihydroartemisinin, and artemisinin plates were freshly prepared by NIMPE using WHO guidelines, and quinine plates were obtained from WHO [10,15,16]. The artemisinin plates were used within three months in the 1998, and 2001 surveys, and within two weeks in 2004/2005 and 2008/9, and all plates were stored at 4°C before use.

Blood was transferred to *in vitro *test plates and incubated for 24 to 36 hours[15]. One of the 12 columns on each plate was used for each patient blood sample. All the wells of the appropriate column were dosed with 50 μl of the blood/medium mixture (BMM, 1: 9). Dosing started with the control (well A) and followed an increasing order of drug concentration ending at well H (the highest concentration). The plates were placed into a candle jar and incubated at 37°C (± 0.5°C) for 24-36 hours. After incubation thick films were made from the pellet in each well of the test plate and stained with 5% Giemsa for 30 minutes. The test was considered valid if 10% or more of the parasites had reached schizont stage (three or more nuclei in the plates) within 24-36 hours. The number of schizonts per 200 parasites was used to assess maturation inhibition. Parasite isolates were classified as sensitive or resistant to a particular drug according to the drug concentration at which schizont maturation was completely inhibited [10,15,17].

### PCR genotyping

Nested PCR was performed as described by Foley *et al *[18] Nested PCR products were analyzed by electrophoresis using 2% agarose for *msp-1 *and *msp-2 *and 1.5% agarose for *glurp*. If the same genotype pattern was found in both samples A and B (i.e. A = primary isolate, B = recurrent isolate), the infection was most likely to be a recrudescence. In contrast, if the patterns of both samples differed, a re-infection was assumed although it is possible that a minor population of the original infection was responsible. It should also be noted that as specific parasite genotypes are highly common in this region, a subject who is infected with a genotype and then cured may be reinfected with the same genotype (and thus misclassified as a recrudescence).

### Data analysis

Mean effective concentrations (EC) were calculated using a computer adapted probit analysis of log-dose responses [17] based on the methods of Litchfield and Wilcoxon [19].

Discrete data were compared using the chi-square test. Comparisons between two independent groups were made using the student's *t *test. Linear regression was used to assess for a trend in parasite clearance times. The correlation coefficients between the parameters of recent treatments were determined by Spearman rank correlation (r).

### Ethics

Consultation was undertaken between the NIMPE team and commune, district and provincial health staff. The team provided patients with information regarding the study and informed consent was documented at the time of enrolment for the surveys. The project was approved by the Human Research Ethics Committee of the National Institute of Malariology, Parasitology and Entomology (Hanoi, Vietnam), the Walter and Eliza Hall Institute of Medical Research (Melbourne, Australia) and Melbourne Health (Melbourne, Australia).

## Results and Discussion

**Figure 2:**
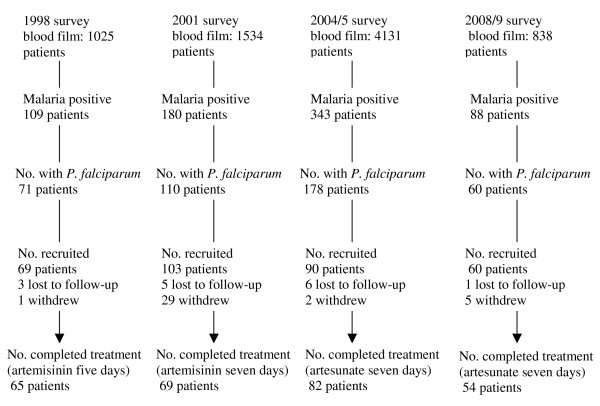
**The number of patients screened for malaria with a blood film at each survey, diagnosed with malaria and *P. falciparum*, recruited into each survey, and who completed a treatment course and follow-up for 28 days is shown**.

The number of patients screened for malaria at each survey, diagnosed with malaria, recruited into each survey, and the number of participants completing the treatment course of artemisinin or artesunate is shown in Figure 2. A total of 65 patients with uncomplicated *P. falciparum *malaria completed a five-day treatment course with artemisinin in the 1998 study, 69 patients completed seven days treatment with artemisinin in 2001, and 82 and 54 patients completed seven days treatment with artesunate in 2004/2005 and 2008/2009 respectively (Table 1).

**Table 1:** Baseline characteristics of study participants and clinical and parasite response to treatment

Characteristics	1998	2001	2004 - 2005	2008 - 2009
Antimalarial drugs ^1^	ART for 5 days	ART for 7 days	AS for 7 days	AS for 7 days
Source of drugs	Vietnam	Vietnam	China	Vietnam
Number of patients	65	69	82	54
Age	22 ± 13	26 ± 13	19 ± 14	30 ± 14
Gender (M/F)	36/29	46/23	61/31	42/12
Temperature (°C)	38.4 ± 1.1	38.6 ± 1.1	38.1 ± 1.2	38.7 ± 1.1
Geometric mean of parasite (Range)	11,200 1,360 - 129,032	13,377 1,120 - 211,200	11,444 1,000 - 200,000	13,942 1,000 - 155,924
Fever Clearance Time (days) Median	1.4 ± 0.5 1 (1 - 2)	1.7 ± 0.7 2 (1 - 4)	1.4 ± 0.6 1 (1 - 3)	1.4 ± 0.5 1 (1 - 2)
Parasite Clearance time (days) Median	1.8 ± 0.9 2 (1 - 3)	2.3 ± 0.9 2 (1 - 3)	2.1 ± 0.7 2 (1 - 3)	1.6 ± 0.7 1 (1 - 3)
No. with recurrent parasitaemia (%)	24 (36.9%)	5 (7.2%)	6 (7.3%)	2 (3.7%)
Treatment failure after genotyping	9 (13.8%)	2 (2.9%)	1 (1.2%)	0 (0%)

1. ART = artemisinin, AS = artesunate

There were differences in mean parasite clearance times between 1998, 2001 and 2004/5 (1.8, 2.3 and 2.1 days respectively, p < 0.01). Artemisinin given for seven days (2001) had a slightly longer parasite clearance time than artesunate for seven days in 2004/5 (2.3 compared with 2.1, p = 0.13) and artesunate given for seven days in 2004/5 had a longer parasite clearance time than in 2008/9 (2.1 compared with 1.6, p < 0.0001) (Tables 1 and 2). However, linear regression analysis revealed that overall there was no significant long-term trend in parasite clearance time in the four surveys.

**Table 2:** Number of *P. falciparum*-infected patients (%) with parasitaemia on days 1-4 of treatment

	1998	2001	2004/05	2008/09
Day 0^1^	65	69	82	54
Day 1	40 (61.5)	29 (42.0)	73 (89)	26 (48.1)
Day 2	10 (15.4)	19 (27.5)	14 (17.1)	5 (9.3)
Day 3	4 (6.2)	6 (8.7)	3 (3.7)	1 (1.9)
Day 4	0	0	0	0

1. Day 0 is day of first diagnosis and commencement of treatment.

The mean parasite clearance times were positively correlated with parasite density at day 0 (r = 0.4; P < 0.001). Overall 36.9% (24/65) patients had recrudescent parasitaemia during the 28-day surveillance period in 1998 after treatment with 5 days of artemisinin. The earliest recrudescence occurred on Day 11. Six patients had a recrudescence between days 11 and 14, 13 patients between days 15 and 21, and five patients between days 22 and 26. Twelve of the 24 patients had a further parasite recrudescence after retreatment with a five-day course of artemisinin (2 occurred between days 11 and 14, 6 between days 15 and 21 and 4 on day 22 or 23). In 2001 the treatment schedule was changed to artemisinin for seven days, and in 2004/2005 and 2008/2009, artesunate was given for seven days. In these surveys, recrudescent parasitaemia was confirmed microscopically in 7.2% (5/69), 7.3% (6/82) and 3.7% (2/54) patients respectively. The treatment failure rates after PCR confirmation were 13.8% (9/65), 2.9% (2/69), 1.2% (1/82), and 0% (0/54), in the four surveys respectively. The proportion of patients with persistent parasitaemia at day 3 was 6.2% in 1998, 8.7% in 2001, 3.7% in 2004/5 and 1.9% in 2008/9 (Table 2). There was no difference in day 3 parasitaemia between the 1998 and 2001 surveys (when artemisinin was used), or between the 2004/5 and 2008/9 surveys (when artesunate was used).

The results of drug susceptibility tests are shown in Table 3. A total of 204 *in vitro *tests were successfully competed for chloroquine, 119 for mefloquine, 89 for quinine, 172 for artemisinin, 81 for dihydroartemisinin and 81 for piperaquine.

**Table 3:** *In vitro *response of *P. falciparum *to anti-malarial drugs in the 1998, 2001, 2004/5, and 2008/9 surveys

Drug	Year	Sample	EC50 (95% CI) ^1^	EC90 (95% CI)	EC 99 (95% CI)
Chloroquine	1998	30	36.7 (27.6 - 48.7)	129 (85.5-194)	360 (196 - 659)
	2001	59	24.4 (19.6 - 30.2)	72.5 (51.2-103)	177 (103 - 302)
	2004/5	34	37.7 (25.8-55.0)	293 (139-617)	1,562 (462 -5,279)
	2008/9	81	67.8 (56.1 - 82.0)	253 (202-358)	735 (535 - 1,272)
Mefloquine	1998	35	83.3 (66.5 - 104)	201 (149-270)	412 (272-622)
	2001	59	42.2 (35.5-50.1)	101 (78.9-129)	205 (143 - 293)
	2004/5	25	60 (42.2 - 85.6)	250 (145-430)	796 (347 - 1,824)
Quinine	1998	31	245 (176-320)	820 (494-1,361)	2,197 (1,018-4,746)
	2001	58	96.3 (78- 119)	277 (199-387)	658 (394-1,098)
Artemisinin^2^	1998	34	30.5 (20.4- 45.5)	201 (111-364)	937 (387 - 2,269)
	2001	58	26.9 (17.2-42.2)	426 (198-914)	4041 (1213-13461)
	2004/5	34	13.7 (8.5 - 22.0)	126 (58.6 - 272)	772 (235-2,537)
	2008/9	46	17.4 (12.0 - 25.3)	92.0 (54.9 - 154)	358 (170 -756)
Piperaquine	2008/9	81	21.1 (16.5-27.1)	62 (91.8-136)	304 (167-554)
DHA	2008/9	81	1.7 (1.4 - 2.2)	14.1 (9-22)	78 (37-163)

1. Values are mean effective concentrations (EC) for *P. falciparum *isolates (number = n) calculated for 50%, 90%, 99% schizont maturation inhibition. Units: nmol/L blood medium mix and 95% confidence intervals.

2. Fresh microtest plates prepared at NIMPE were used in the artemisinin experiments. These were replenished in the field every three months in 1998 and 2001, and every 2 weeks in 2004/5 and 2008/9

The mean EC_50 _for artemisinin was 30.5 nmol/L in 1998, 26.9 nmol/L in 2001, 13.7 nmol/L in 2004/5 and 17.4 nmol/L in 2008/9. There was a 4-fold increase in the EC_99 _over the three year time period 1998 to 2001, (P < 0.001) but no difference between 1998 and 2008/9 (p = 0.127), and no difference between 2004/5 and 2008/9 (p = 0.511).

This study examined *P. falciparum *resistance patterns over 10 years in infected patients treated with artemisinin or artesunate in two sites in Phuoc Long district, Binh Phuoc province, Vietnam, which were thought to be likely sites for emerging resistance to artemisinin due to the length of time that artemisinin-based compounds had been in use. Seven day treatment with artesunate was found to be effective in this area in 2008/9 with a recrudescence rate from 0% to < 5% and no cases of early treatment failures. There were no significant differences in fever or parasite clearance times between the surveys in1998 and 2008/9. Treatment failure rates reduced during the period. These findings were supported by *in vitro *testing of *P. falciparum *isolates, although tests in 2001 raised the possibility of reduced sensitivity to artesunate.

In the 1998 survey, patients were treated with five days of monotherapy with artemisinin compared to treatment with artemisinin or artesunate for seven days in later surveys. The recurrent parasitaemia rate of 36.9% and treatment failure rate of 13.8% confirms the lack of efficacy of this approach[20]. Artesunate was used in the last two surveys and showed improved parasite clearance and reduced treatment failure rates compared to the first two surveys, which used artemisinin. However, the surveys were conducted at two different but nearby communes, and it is possible that the two sites had different malaria drug resistance patterns.

The reduced sensitivity to artemisinin in *in vitro *tests in the 2001 survey raised the possibility of increasing *P. falciparum *tolerance to artemisinin, although the significance of reduced parasite sensitivity in EC90 and EC99 tests is not clear. Menard *et al *[21] found that 55 non-immune patients living in Bangui, Central African Republic, had parasitological cure rates of 100%, 95%, and 85% on days 14, 28, and 42, respectively. Although there were no significant differences in parasitaemia density, 50% inhibitory concentration of dihydroartemisinin, and frequency of variant MDR1 codon 86 between patients who were cured and those who displayed recrudescence, the 90% inhibitory concentration for dihydroartemisinin and the number of genotypes isolated were both higher in the recrudescent patients (five- and two-fold, respectively).

A more likely explanation for the increase in EC90 and EC99 in the 2001 survey was deterioration of artemisinin plates during the three months they were kept in the field during the survey. To overcome this possibility plates were replenished every two weeks in the 2004/5 and 2008/9 surveys. However, the reason for prolonged fever clearance time and the higher day three parasitaemia in the 2001 survey is not clear and the possibility remains that the parasites circulating at the time were more tolerant to artemisinin than in the earlier survey. The use of artesunate for treatment in the 2004/5 and 2008/9 surveys, and the introduction of ACT (CV8) as routine treatment in health centres in this district from 2003 may have decreased the chance of drug resistant *P. falciparum *infection being detected in the later surveys.

*In vitro *resistance of *P. falciparum *field isolates to artemether and corresponding point mutations in the SERCA-type PfATPase6 have been reported in French Guiana and Senegal [22]. Also, artemisinin resistance has recently been described on the Cambodia-Thai border [4] and in Cambodia [5]. These studies confirmed the existence of *P. falciparum *resistance to artesunate in the Mekong region although treatment failures with ACT remain low.

The limitations of this study included the use of different formulations and treatment regimes with artemisinin and artesunate in the four surveys. However, the design of each survey was consistent with WHO protocols, and treatment of patients was consistent with national malaria treatment guidelines that were current at the time. The first two surveys were also conducted at a different commune than the last two surveys. The two communes were about 80 Km apart and although malaria endemicity is similar at the two sites, it is possible that different patterns of drug resistant malaria emerged at each site during the study period. Anecdotal evidence from a nearby district has suggested higher levels of persistent parasitaemia on day 3, and this is currently being investigated (NV Thanh, personal communication). Another weakness was that although an adequate sample of more than 50 patients was recruited in each survey not all parasite isolates were able to be established in culture for *in vitro *testing. There was also considerable loss to follow-up in the 2001 survey. This and the non-random selection of patients may have biased the results.

The main strengths of the study included the long follow-up period over 10 years and the careful manner in which the surveys were conducted. For each survey, the same experienced national team was involved in recruiting, treating and monitoring patients, who were given directly observed therapy and monitored closely for the first seven days. Artemisinin plates were replenished in the field every three months in the first two surveys and every two weeks in the last two surveys reducing the likelihood of plate deterioration.

## Conclusions

This study has shown relatively stable levels of *P. falciparum *sensitivity to artemisinin compounds over a ten-year period in two communes in Phuoc Long district, Binh Phuoc province, Vietnam. The introduction of ACT in 2003 may have protected against the spread of artemisinin resistance in this area. Adherence to the latest WHO and Vietnamese malaria treatment guidelines, which recommend ACT as first line therapy in all malarious areas, and consistent malaria control measures such as early microscopic diagnosis, drug resistance monitoring, provision and use of bed nets, and health worker training will be essential if the development of resistance to artemisinin-based compounds is to be prevented in Vietnam.

## Competing interests

The authors declare that they have no competing interests.

## Authors' contributions

NVT was responsible for planning, and implementation of the study and analysis of results, and preparation of the first draft of the manuscript in collaboration with other authors. AC provided training for NVT, supervision of the laboratory aspects of the project, and had input into the manuscript. NTT provided supervision for the NIMPE and provincial field team who implemented the project, and had input into data analysis. GC provided ongoing supervision in Hanoi on behalf of UOM, and had input into the manuscript. BP and TT had primary involvement in the implementation of field and laboratory aspects of the project. NH provided overall supervision of NIMPE staff and was involved in planning the project. BB was involved in planning the project, procurement of funding, supervision, and writing the manuscript.

All authors read and approved the final manuscript.
